# Exploring drivers and challenges influencing antibiotic prescribing in outpatient settings and possible mitigation strategies in the United Arab Emirates: a qualitative study

**DOI:** 10.1093/jacamr/dlad109

**Published:** 2023-10-09

**Authors:** Ahmed A Sadeq, Farah Ahmed Issa, Mina Bakhit, Maitha Abdul-Aziz Al-Tamimi, Zahir Osman Eltahir Babiker, Raghad S Ismail Alshabebi, Jehad Abdallah, Emmanuel Fru Nsutebo, Marleine B Moukarzel, Rawan Abukhater, Barbara R Conway, Stuart E Bond, Sidra Khan, Mamoon A Aldeyab

**Affiliations:** Department of Pharmacy, Shaikh Shakhbout Medical City in Partnership with Mayo Clinic, Abu Dhabi, PO BOX 11001, United Arab Emirates; Department of Pharmacy, School of Applied Sciences, University of Huddersfield, Huddersfield HD1 3DH, UK; Department of Medicine, Shaikh Shakhbout Medical City in Partnership with Mayo Clinic, Abu Dhabi, PO BOX 11001, United Arab Emirates; Institute for Evidence-Based Healthcare, Bond University, Gold Coast, QLD 4226, Australia; Department of Pharmacy, Shaikh Shakhbout Medical City in Partnership with Mayo Clinic, Abu Dhabi, PO BOX 11001, United Arab Emirates; Division of Infectious Diseases, Shaikh Shakhbout Medical City in Partnership with Mayo Clinic, Abu Dhabi, PO Box 11001, United Arab Emirates; Department of Intensive Care, Shaikh Shakhbout Medical City in Partnership with Mayo Clinic, Abu Dhabi, PO BOX 11001, United Arab Emirates; Infectious Disease Department, Al Rahba Hospital, Abu Dhabi Health Services (SEHA), Abu Dhabi, United Arab Emirates; Division of Infectious Diseases, Shaikh Shakhbout Medical City in Partnership with Mayo Clinic, Abu Dhabi, PO Box 11001, United Arab Emirates; Department of Pharmacy, Shaikh Shakhbout Medical City in Partnership with Mayo Clinic, Abu Dhabi, PO BOX 11001, United Arab Emirates; Department of Medicine, Shaikh Shakhbout Medical City in Partnership with Mayo Clinic, Abu Dhabi, PO BOX 11001, United Arab Emirates; Department of Pharmacy, School of Applied Sciences, University of Huddersfield, Huddersfield HD1 3DH, UK; Institute of Skin Integrity and Infection Prevention and Department of Pharmacy, School of Applied Sciences, University of Huddersfield, Huddersfield HD1 3DH, UK; Department of Pharmacy, School of Applied Sciences, University of Huddersfield, Huddersfield HD1 3DH, UK; Department of Pharmacy, Mid Yorkshire Hospitals NHS Trust, Wakefield WF1 4DG, UK; Department of Pharmacy, School of Applied Sciences, University of Huddersfield, Huddersfield HD1 3DH, UK; Department of Pharmacy, School of Applied Sciences, University of Huddersfield, Huddersfield HD1 3DH, UK

## Abstract

**Objectives:**

Healthcare institutions implement antimicrobial stewardship (AMS) programmes to optimize the use of antibiotics. The focus is often on inpatient rather than outpatient settings. We aimed to explore perceptions of AMS stakeholders on effective interventions for appropriate antibiotic use in outpatient settings, and the role of clinical pharmacists in the AMS multidisciplinary team.

**Methods:**

A qualitative semi-structured interview study using thematic analysis by two researchers independently. Participants that practice AMS programmes were recruited from healthcare facilities in the United Arab Emirates (UAE). Interviews were conducted face to face or online and transcribed verbatim.

**Results:**

Four themes emerged: (i) Perceived factors leading to unnecessary or inappropriate antibiotic prescribing and their impact on patients and the community; (ii) current outpatient AMS activities and perceived barriers and facilitators for their sustainability; (iii) suggested outpatient AMS strategies to be implemented in outpatient settings; and (iv) perceived future AMS implementation barriers and suggested mitigation strategies.

**Conclusions:**

Several AMS interventions, together with the presence of a clinical pharmacist, may be effective in improving antibiotic use in UAE outpatient settings. Future research should investigate the most appropriate AMS strategy considering barriers and possible mitigation strategies to ensure sustainability.

## Introduction

Antibiotic-resistant bacteria have become more prevalent globally over the past few decades,^1,2^ with consequent infections resulting in extended hospital stays, expensive treatments and greater fatality rates.^3–6^

Healthcare institutions or departments (e.g. ICUs) implement antimicrobial stewardship (AMS) programmes to optimize the use of antibiotics in order to improve patient outcomes, reduce side effects (e.g. antibiotic resistance and toxicity) and provide cost-effective treatment.^7–11^ Those programmes with a dedicated infectious disease clinical pharmacist, who is actively involved in antibiotic management, have resulted in higher adherence to advised antibiotic therapy procedures than those relying on ward pharmacists.^12^

The focus of many AMS programmes has been to optimize the use of antibiotics in hospitalized patients^7,10,13–19^ and emergency departments^20–24^ rather than hospital outpatient settings, leading to a dearth of research in such a setting. A recently published meta-analysis highlighted experimental studies testing AMS interventions in hospital outpatient settings, and the authors found that AMS can improve several outcomes, including antibiotic consumption and adherence to protocols.^25^ However, there is still a gap regarding which AMS strategy could be successfully implemented in hospital outpatient settings. In the United Arab Emirates (UAE), no studies have qualitatively explored clinicians’ perspectives, attitudes and behaviours towards antibiotic prescribing and AMS strategies in the outpatient setting.

This study aimed to explore healthcare professionals’ (HCPs’) perceptions on AMS strategies in the outpatient setting in UAE, the role of clinical pharmacists in the AMS multidisciplinary team, and AMS implementation challenges and possible mitigation strategies.

## Methods

### Design

This qualitative study used semi-structured interviews. The consolidated criteria for reporting qualitative research (COREQ) checklist was used in this study.^26^

### Participants and recruitment

Participants were recruited from healthcare facilities in the UAE that use AMS programmes. They had various roles, including working in outpatient settings.

Participants were recruited between 24 November 2022 and 10 December 2022. Through e-mail communication, gatekeeper approval was obtained from hospitals’ and facilities’ education and quality departments with dedicated AMS programmes to disseminate the information to their clinicians in the form of an open invitation, followed by snowballing, where the participants recommended other clinicians to participate in the study. Participants who responded to the invitation signed written consent forms.

### Procedure

Draft interview questions were based on the results and conclusions of the findings of a recent meta-analysis on effective AMS interventions in outpatients,^25^ along with inputs from experts in the field. The final questions were agreed upon by consensus amongst five authors (A.A.S., M.T., F.I., M.B., M.A.A.). After piloting with four participants, who were not included in the study, further minor amendments were incorporated (the question guide is available as Appendix 1, available as Supplementary data at *JAC-AMR* Online).

Interviews were conducted face to face or online using Microsoft Teams^®^ by two authors (A.A.S. and M.T.). Interviews were recorded either through the Microsoft Teams recording option, if the interview was conducted online, or through an external device, if the interview was conducted face to face. Interviews were conducted in English, primarily spoken by HCPs in the UAE. Although the participants were aware of the study goals, they had no prior knowledge of the questions posed during the interview.

### Data analysis

After 22 participants had been interviewed, a preliminary thematic analysis was undertaken. It was decided that data saturation^27^ had occurred, and no further recruitment of participants was required. Data saturation was defined as when no new ideas or constructs emerged from two consecutive interviews. Audio-recorded interviews were transcribed verbatim and independently analysed by two authors (A.A.S. and F.I.). Transcripts were thematically analysed using the process outlined by Braun and Clarke.^28^ After familiarizing themselves with the interview transcripts, the analysts generated overarching themes and subthemes. The analysis was a data-driven process that was partially inductive in nature. Authors compared and discussed their themes and analyses and, with the inputs of two additional authors (M.A.A., M.B.), came to a consensus. All authors then agreed to the final themes and quotes.

There was no identification of the participants following the interview. Transcripts and all audio recordings were coded and saved to the University of Huddersfield’s One Drive^®^. Only the authors of this study had access to the drive, and all audio recordings were erased after transcription.

### Ethics

Ethics approval was provided by Shaikh Shakhbout Medical City (SSMC), where the study was conducted (reference number: SSMCREC-342). The University of Huddersfield also approved the study (reference number: SAS-SRIEC-21.12.22-2).

## Results

**Figure 1.**
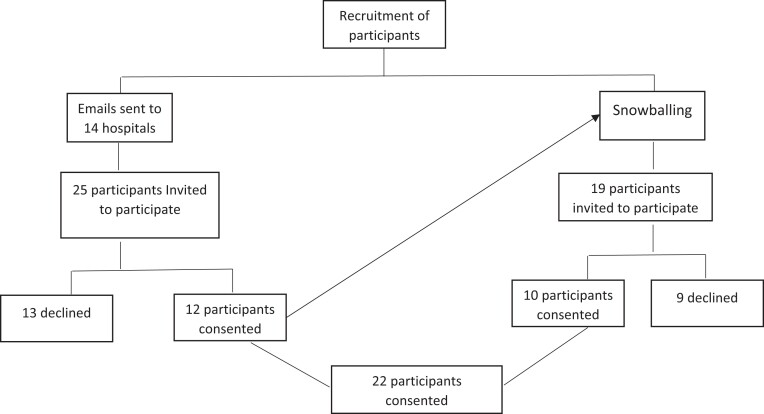
Sampling strategy.

Fourteen hospitals across the UAE were approached, and 12 participants consented. Consenting HCPs referred us to a further 19, of whom 10 consented. The most common reason for declining participation was insufficient time to be interviewed. A total of 22 participants were interviewed and the cumulative duration of the interviews was 10.3 h. Figure 1 describes the sampling strategy.

Five participants were internal medicine physicians, 11 were infectious disease physicians and 6 were clinical pharmacists, summarized in Table 1, along with details of the healthcare facility.

**Table 1.** Demographics of healthcare facilities (HCFs) and participants

City	Number of HCFs	Number of HCPs
Hospital demographics		
Abu Dhabi	6 hospitals and 1 primary care clinic	17
Al Ain	1 hospital	1
Dubai	3 hospitals	3
Sharjah	1 hospital	1
Specialty		Number of HCPs
Participant demographics		
Internal medicine		
Consultant		4
Specialist		1
Resident		1
Infectious disease		
Consultant		8
Specialist		2
Pharmacy		
Clinical pharmacist		6
Gender		
* *Male		14
* *Female		8
Age (years)		
* *25–39		6
* *40–59		16
Experience (years)		
* *<5		3
* *5–9		5
* *10–14		8
* *≥15		6

HCF: health care facility; HCP: health care professional

Table 2 summarizes the four main themes and subthemes discussed below.

**Table 2.** A summary of the identified themes and subthemes

Theme	Subthemes		
Theme 1: Perceived factors leading to unnecessary or inappropriate antibiotic prescribing and their impact on patients and the community	Perceived clinician-related factors:	Perceived patient-related factors:	Perceived harms of inappropriate antibiotic prescribing:
	Time pressure effect on antibiotic prescribing Perceived patient demand for antibiotics Concerns about lack of satisfaction with service if antibiotics are not prescribed Loss of patients’ follow-up to de-escalate or change antibiotic therapy according to culture results Lack of unified outpatient antimicrobial guidelines	Patients’ resistance and not convinced that their illness is self-limiting Easy access to antibiotics over the counter	Cost of antibiotic therapy Side effects Resistance caused by antibiotic prescribing or not completing antibiotic treatment, or unnecessary use of antibiotics by patients

Theme 2: Current outpatient AMS activities and perceived barriers and facilitators for their sustainability	Current AMS strategies:	Prescriber-related challenges for sustaining AMS strategies in outpatient settings:
	Lack of effective AMS activities in the outpatient settings More focus on inpatient rather than outpatient settings Reminding physicians about the appropriate use of antibiotics Prospective audit and feedback Use of phone applications	Prescriber’s resistance to applying updated practices Variations in physicians’ practices Lack of awareness regarding new prescribing guidelines Time pressures

Theme 3: Suggested outpatient AMS strategies to be implemented in outpatient settings	Policy and regulatory strategies:	Organizational-level strategies:	Clinical strategies:
	Prospective/retrospective audit and feedback Antibiotic restriction policy Availability of unified guidelines alligned with local antibiogram	The need for dedicated AMS staff such as clinical pharmacists and infection control specialists Commitment from different stakeholders Leadership support to implement AMS activities	Providing education for HCPs about AMS, antibiotics, antibiograms and AMR Implementing delayed antibiotic dispensing Patient education about AMR Use of CDSS such as order-sets, system alerts, and phone applications

Theme 4: Perceived future AMS implementation barriers and mitigation strategies	Barriers to implementing antibiotic restriction policy:	Barriers to recruiting dedicated AMS staff:	Barriers to using antibiotic delayed dispensing:	Suggested mitigation strategies:
	Impacting workflow Stealing prescribers’ autonomy	Lack of resources Staff workload	Patients’ behavioural culture and non-compliance Unstable medical conditions	Dedicated AMS staff Conducting audit activities Gap analysis Presence of a clinical pharmacist

### Theme 1: perceived factors leading to unnecessary or inappropriate antibiotic prescribing and their impact on patients and the community

#### Perceived clinician-related factors

Most participants believed that time pressure, and patient dissatisfaction if antibiotics were not prescribed are the main clinician-related factors leading to inappropriate antibiotic prescribing in the outpatient setting.

‘*Time is also a challenge that is there. Even if the guidelines are there, they may not be committed to [them] because there is not enough time to explore them’ PI01*

*‘Pressurizing of the physician by the patient about having the antibiotic. Some physicians might worry about losing satisfaction of the patient’ PI04*

Some participants highlighted that lack of patient follow-up and communication post intervention limited opportunities for antibiotic de-escalation. Consequently, it was difficult to change antibiotic therapy following bacterial culturing in the outpatient setting.

‘*So I believe we need to order bacterial culture to de-escalate these type of antibiotics according to the culture and we [prescribers] need the patient to follow up on time’ PI03*

#### Perceived patient-related factors

Clinicians highlighted some patient-related factors associated with inappropriate antibiotic prescribing, including patients not convinced that antibiotics are not needed for self-limiting conditions, and the wide accessibility of over-the-counter antibiotics that can be acquired without a medical prescription.

*‘Patient or family insistent on using antibiotics even for viral infections, as some people can get antibiotic from outside without medical prescription’ PI20*

#### Perceived harms of inappropriate antibiotic prescribing

Participants noted possible harms arising from inappropriate antibiotic prescribing, including side effects, the cost of therapy, and antibiotic resistance.

‘*Cost of antibiotics in outpatient [setting] could be challenging sometimes if the patient has no insurance’ PI19*.

*‘It is a global thing, and it is our [HCPs’] responsibility in the first place to fight against the spread of resistance against antimicrobials and save the current available ones for the community and to enhance the awareness in the community itself’ PI09*

### Theme 2: current outpatient antimicrobial stewardship activities and perceived barriers and facilitators for their sustainability

#### Current AMS strategies

All participants reported a lack of awareness of practical AMS activities in the outpatient setting, which extended beyond their current inpatient setting. Participants highlighted that current stewardship activities are focused on inpatient rather than outpatient settings.

*‘In our hospital, in terms of stewardship, we don't have anything specific as such, but we have more activities in the inpatient setting rather than the outpatient’ PI15*

Several ongoing AMS strategies were mentioned in outpatient settings, including sending reminders about appropriate antibiotic prescribing, audits and feedback, key performance indicators (KPIs) monitoring, and implementing mobile applications that advise clinicians on first-line antibiotic choice.

‘*Challenges will be that you need to keep reminding physicians about the appropriate use of antibiotics in order to sustain what we are trying to achieve out of the education process’ PI19*

*‘We [in the hospital] have KPIs for stewardship including the percentage of the proper antibiotic selection, and the proper microbiological work up before the initiation of restricted antibiotics’ PI22*

The lack of patient follow-up and lack of adherence to the implemented hospital local antimicrobial guidelines by physicians hindered the sustainability of AMS activities in outpatient settings.

‘*Some of the doctors, either they don’t have time to follow up on those results [cultures] with the patient, or the patient will disappear and it is hard to reach that patient to discuss the results’ PI22*

*‘There is lack of adherence to guidelines’ PI17*

#### Prescriber-related challenges for sustaining AMS strategies in outpatient settings

Participants mentioned several challenges hindering the sustainability of AMS activities in outpatient settings. Most participants highlighted clinicians’ resistance to change their antibiotic prescribing behaviour, lack of awareness regarding new guidelines, variations in their background practices, and time pressure as the most common challenges.

‘*Doctors’ resistance, because some of them think we are interfering with the decision-making process, so their autonomy and control are affected’ PI07*

*‘The most important challenge is to fight with what physicians have learned from the past, and they are used to do’ PI18*

### Theme 3: suggested outpatient AMS strategies to be implemented in outpatient settings

Multiple AMS strategies have been suggested by the participants to improve antibiotic prescribing in outpatient settings.

#### Policy and regulatory strategies

Strategies considered by participants for reducing injudicious antibiotic prescribing include regular audits and feedback sent to high antibiotic-prescribing clinicians, and monitoring process of antibiotic prescribing with data linkage between antibiotic prescribing and reported future side effects.

*‘It is a very good idea to annually conduct retrospective audits’ PI10*

*‘We measure those activities before and after the intervention and monitor them to what extent they are effective’ PI14*

Other strategies advocated by some participants were implementing antibiotic restriction policies and the provision of institution-specific antimicrobial guidelines integrating a local antibiogram, which may standardize antibiotic practices among the different clinicians in outpatient settings.

‘*Actually, restricting antibiotics in outpatient setting will have a good impact, and in my opinion this kind of restriction should be done in a manner of not being fully restricted but keep it restricted for specific diseases’ PI09*.

*‘The long-term impact of the local antimicrobial guidelines is to reduce resistance, this is a top priority. Then, reducing the collateral damage associated with antibiotics’ PI04*

*‘To know your local antibiogram and to know your local resistance pattern, so you are doing the right choice’ PI06*

#### Organizational-level strategies

A few participants highlighted organizational-level strategies that can be implemented such as having dedicated AMS staff within outpatient settings. Also, a commitment from all stakeholders and having leadership support to implement AMS activities were considered to be of utmost importance.

*‘I think if we [prescribers] have pharmacists, infection control specialists, and infectious disease consultants on board who work in good collaboration with labs, I think that helps a lot’ PI11*

*‘Yes, there is commitment from our team [prescribers]. It is done because we frequently receive reminders regarding antimicrobial therapy’ PI03*

Clinical pharmacists were seen as the ‘cornerstone’ for AMS programmes in outpatient settings and may help to oversee how antibiotics are used, in addition to providing education and ensuring that antibiotics are prescribed for the right indication.

‘*Clinical pharmacists in the outpatient are very essential, and that will support the programme because they can educate and they can teach providers about antibiotics and guidelines’ PI22*.

*‘Of course, they [pharmacists] are the cornerstone of antimicrobial stewardship programme, and they monitor antibiotics that are being prescribed, so they make sure dose is right and the indication is right, especially those [pharmacists] who have an infectious disease interest’ PI21*.

#### Clinical strategies

Participants highlighted clinical strategies that may be introduced into hospital outpatient settings, including educating HCPs about AMS and appropriate antibiotic use, the most common conditions, their aetiologies, and whether an antibiotic prescription is needed.

*‘To start with raising awareness like campaigns to educate physicians about prescribing in outpatient settings and how to use antibiotics appropriately’ PI19*

*‘PI06*

Another clinical strategy advocated by some of the participants as successful was the use of delayed dispensing. However, a few participants commented that it should be applied only when relevant.

*‘Sometimes you can delay depending on the patient condition so you send the patient home and tell him do not dispense your prescription yet and you can pick it up later’ PI06*

Clinicians were also encouraged to provide outreach education and awareness to the general public about infections and antibiotic resistance, which helps for the correct use of antibiotics by the community.

‘*I mean (education) for the patient, nurse, and physician. So, for example, when the patient comes to the outpatient clinic, some kind of orders are by demand rather than by true infections’ PI07*.

Most participants recommended using a clinical decision support system (CDSS) as a tool that can support clinicians in using antibiotics effectively. Electronic healthcare system alerts appearing when choosing an inappropriate antibiotic, displaying patient history in terms of microbiological cultures and admissions, using automated order-sets, and using accessible phone applications can support CDSS utilization.

‘*I think the application that we have [in the hospital] called ‘Firstline^™^’ is really a useful tool to use, particularly as [antibiotic] regimens change every time and it is hard for us to keep up with them, and some of those can be old fashioned’ PI10*.

*‘It will be useful to implement electronic system order-set similar to inpatient setting, so it is good to have order-sets in outpatient setting for common conditions that we [prescribers]encounter’ PI15*.

*‘Electronic medical record (CERNER^®^) has an alert that pops up in the system for sensitive antibiotics or serious infections. The pop-up alerts for duration of antibiotics usually appears when the duration is about to end’ PI12*.

### Theme 4: perceived future AMS implementation barriers and suggested mitigation strategies

#### Barriers to implementing antibiotic restriction policy

Many participants regarded restricting antibiotics in the outpatient setting as a way of reducing the use of certain antibiotics, such as broad-spectrum antibiotics, and decreasing antibiotic resistance. However, doing so could impact workflow in such settings and possibly frustrate some clinicians as they may feel like they are losing their autonomy in prescribing antibiotics.

‘*Restriction policy helps in appropriate antibiotic use’ PI13*.

*‘Doctors’ resistance, because some of them think we are interfering with the decision-making process, and [their] autonomy and control are affected, which is not true’ PI07*

#### Barriers to recruiting dedicated AMS staff

The availability of resources was considered crucial to support regular stewardship activities. Staff with AMS responsibilities may face time pressure, which may sometimes hinder some AMS activities from being conducted regularly, such as audits, ongoing communication with physicians, and drafting or updating guidelines.

‘*There should be an automatic reporting system or I.D. staff or dedicated person who should have basic training in common infectious diseases and have some communication skills and a good reputation on working physicians’ PI07*

*‘Challenges to make guidelines or review every prescription definitely needs time, needs effort, so I think the main challenge is protected time’ PI13*

#### Barriers to using delayed antibiotic dispensing

Participants felt that patients will never comply with the advice when delayed antibiotic dispensing is applied, and may dispense the prescribed antibiotic for themselves or give it to family members.

*‘So, I do not think if you dispense the medication and you tell the patient to wait for two or three days until I call you, the patient will follow your instruction, instead, they [the patients] will start the antibiotic once they leave your facility’ PI22*

*‘The problem is, I don't know with our behavioural culture, so if you give me a prescription, I may not take it, but I may give it to other family members, so I don’t think it is a good idea’ PI21*

#### Suggested mitigation strategies

Participants proposed a number of mitigation strategies to help AMS implementations in the outpatient setting. They highlighted the need for a monitoring process to assess the impact of antibiotic prescribing, ensuring gap analysis for effective restriction policy, and the presence of clinical pharmacists as facilitators for appropriate implementation of AMS strategies.

*‘But once you are doing that and setting up this you need to make sure that there is a process of how you are going to monitor that prescribing behaviour or impact of prescribing those antibiotics and possible side effects, and if the patient is coming up with any complication’ PI06*

*‘Clinical pharmacists have a big role in antimicrobial stewardship. I believe clinical pharmacists have the knowledge and they can review all prescriptions especially the ones that are not prescribed by ID physician’ PI13*

## Discussion

This study interviewed clinicians who practise in outpatient settings and work in UAE healthcare facilities that adopt AMS programmes. Therefore, their views and perceptions are vastly beneficial and can be considered as the building blocks to implement AMS in outpatient settings.

Time pressure was one of the challenges faced by the prescribers, affecting compliance with guidelines, similarly reported in three other qualitative studies that were conducted within the outpatient setting in multiple European countries and Canada.^29–31^ Other challenges were the feeling of pressure from patients and their demands for antibiotics to be prescribed, and worries associated with patient dissatisfaction, which were also findings shared in several qualitative studies.^30,32–34^

Most of the participants reported a lack of AMS activities in the outpatient setting of their respective facilities, a finding also reported in two other studies conducted in the USA.^35,36^ A few participants mentioned AMS activities that are currently taking place in their facilities, including prospective audit and feedback, which proved to improve AMS in outpatient settings,^37–40^ and the use of phone applications integrating hospital guidelines, which has also been shown to be effective.^41–43^ Concerns were raised regarding sustainability of these activities, hindered by certain prescribers’ resistance to change, lack of updates about new guidelines, and variations in practice. This was also a finding in a qualitative study conducted in the USA, so it is not unique to this country.^30^

Several AMS strategies were suggested by participants to improve antibiotic use in the outpatient setting. For example, antibiotic restriction policies, which have been reported to be effective elsewhere in the UK and in the USA.^44,45^ In addition, unified institution-specific antimicrobial guidelines were considered beneficial, given the variations in antibiograms between different cities and countries.^7^

Another suggested strategy by the participants was to secure leadership support within organizations to provide dedicated staff to support AMS activities. This strategy was also proposed in one qualitative study in Canada.^30^ Participants also recommended seeking leadership support in granting commitment to AMS from all stakeholders in healthcare facilities, and this was found to be effective in one study that involved five primary care clinics in the USA.^40^

Recommended clinical strategies included education for patients and HCPs on antibiotics, which proved to be an effective strategy in both inpatient and outpatient settings in other countries.^46–48^ A CDSS has also been found to have a positive impact on improving AMS in outpatient settings.^49^ Although there were contradictory opinions amongst the participants about the benefits of employing a delayed antibiotic dispensing strategy, it has been found to be effective in decreasing antibiotic use in a systematic review.^50^

Barriers to implementing some of the suggested strategies were discussed by the participants, who believed that restricting certain antibiotics would impact workflow and may be perceived by prescribers as a potential disruption of their autonomy, which was the case in one study conducted in a paediatric hospital.^51^ This study was also able to explore mitigation strategies for the identified barriers, with participants emphasizing the importance of the presence of clinical pharmacists as an effective part of any AMS programme, a finding that was supported by multiple studies.^52–54^

Our study has a few limitations. Firstly, there was not an even distribution of participants across all specialities, but this was expected as participation was voluntary. Secondly, the generation of themes was dependent on the personal understanding of participants’ responses; however, two authors conducted the data analysis separately and a further two agreed on the final themes by consensus. Lastly, this study mainly specifies the UAE outpatient settings, and we are unsure about the generalizability to other settings.

The participants in this study gave their insights on possible interventions that could enhance antimicrobial stewardship in the outpatient setting. Given the fact that antimicrobial stewardship in outpatient settings is lacking in UAE, as highlighted by the participants, and the uncertainty about the possibility of adopting successful AMS inpatient interventions in the outpatient settings, this study has explored strategies to enhance AMS, specifically in the outpatient setting. Therefore, it can form the basis for further improvement of antibiotic prescribing and utilization within such settings. Findings generated from this study can be implemented and evaluated for their effectiveness in future studies concerning UAE outpatient settings.

### Conclusions

Inappropriate use of antibiotics has increased the need for AMS in the outpatient setting. There are several challenges to properly execute AMS in such settings and consequently interventions were recommended to improve antibiotic use in outpatient settings. Several barriers may hinder the sustainability of those interventions, therefore mitigation strategies should be in place.

## Supplementary Material

dlad109_Supplementary_Data

## Supplementary data

Appendix 1 available as Supplementary data at *JAC-AMR* Online.
