# Supplementary material for: Exploring drivers and challenges influencing antibiotic prescribing in outpatient settings and possible mitigation strategies in the United Arab Emirates: a qualitative study
Source: JAC Antimicrob Resist. 2023 Oct 9;5(5):dlad109. doi: 10.1093/jacamr/dlad109 (PMC10561535; doi:10.1093/jacamr/dlad109)
Supplement: dlad109_Supplementary_Data [file dlad109_supplementary_data.docx]

Appendix 1: Interventions for improving antibiotic use in outpatient settings

Interview questions

**Interview topic guide**

A series of interview questions are given in this paper. This topic guide may change over time, both before and during the study, in response to new insights. Extra questions might be asked to check participant understanding and to clarify responses.

**Baseline questionnaire**

*To begin with, we have a few questions about you. For each, I will read the question and the response options, and you can indicate which apply.*

**First, how old are you?**

[ ] 18-39 years [ ] 40-59 years [ ] 60 years and over

**What is your gender?**

[ ] Female [ ] Male [ ] Prefer not to say

**What is your specialty at the hospital?**

**For how many years have you worked as a [reported speciality]?**

[ ] Less than 5 years [ ] 5 – 9 years [ ] 10 – 14 years [ ] 15 years or more

**Interview questions**

*Thank you for agreeing to participate in this interview. For the next few questions, I would like your views on interventions for improving antibiotic use in outpatient settings. There are no right or wrong answers. I am just interested in your perceptions and views.*

1. Firstly, can you please tell me what the term antimicrobial stewardship means for you, and why some see it as a useful approach?
2. Can you tell me what antimicrobial stewardship activities are currently being conducted in the outpatient setting in your hospital?
3. Do you believe that those mentioned activities are effective? And why? Why not?
4. What other intervention(s), do you think, can lead to appropriate antibiotic use in outpatient settings?
5. What are the challenges that may arise while implementing those interventions?
6. Can you share a successful antimicrobial stewardship activity that been recently introduced this year? How do you measure its effectiveness? Any barriers faced during the implementation? How did you overcome those barriers?
7. Do you think there is a commitment from stakeholders towards antimicrobial stewardship activities? How about you, do you feel committed to antimicrobial stewardship? Why and why not?
8. Is there sufficient support received from your hospital for the implementation of antimicrobial stewardship activities?
9. What are the challenges that could jeopardize the appropriate use of antibiotics in hospitals generally? And specifically in the outpatient setting?
10. Do you think health care professional education is an effective intervention? And why/why not?
11. What impact could implementation of local hospital antimicrobial guidelines have on antibiotic use?
12. Is it feasible to conduct regular retrospective audit and feedback in the outpatient setting? Are there enough resources?
13. How can technologies help in increasing appropriateness of antibiotic use in the outpatient setting? Can you give an example?
14. Do you think restricting certain antibiotics can be an effective intervention in the outpatient setting? What impact could It have on clinical prescribing workflow?
15. Delayed antibiotic prescriptions are being used in some countries in Europe. Do you think this is applicable in your outpatient clinic? And would it decrease antibiotic use while maintaining appropriate patient treatment?
16. How can clinical pharmacists contribute to an antimicrobial stewardship program? What part do you believe a clinical pharmacist may play in enhancing the outpatient setting's usage of antibiotics?
